# Autophagic clearance of bacterial pathogens: molecular recognition of intracellular microorganisms

**DOI:** 10.3389/fcimb.2013.00054

**Published:** 2013-09-30

**Authors:** Maria Eugenia Mansilla Pareja, Maria I. Colombo

**Affiliations:** Laboratorio de Biología Celular y Molecular, Facultad de Ciencias Médicas, Instituto de Histología y Embriología-CONICET, Universidad Nacional de CuyoMendoza, Argentina

**Keywords:** autophagy, phagocytosis, intracellular pathogens, recognition, xenophagy

## Abstract

Autophagy is involved in several physiological and pathological processes. One of the key roles of the autophagic pathway is to participate in the first line of defense against the invasion of pathogens, as part of the innate immune response. Targeting of intracellular bacteria by the autophagic machinery, either in the cytoplasm or within vacuolar compartments, helps to control bacterial proliferation in the host cell, controlling also the spreading of the infection. In this review we will describe the means used by diverse bacterial pathogens to survive intracellularly and how they are recognized by the autophagic molecular machinery, as well as the mechanisms used to avoid autophagic clearance.

## Intracellular pathogens: different survival strategies

Certain pathogens invade host cells to be protected from systemic immunity. However, these microorganisms face the challenge posed by intracellular innate defense responses designed to eliminate the invader. Therefore, once in the host cell, pathogens use sophisticated strategies to avoid destruction. These strategies comprise: (i) lysis and escape from the phagosome/vacuole; (ii) modification of the phagosomal compartment; (iii) survival in acidic/degradative compartments. Even though, this classification it is useful, it is not strict and some microorganisms may use more than one strategy to actually survive in the host cell. In this review we describe selected bacterial pathogens that belongs to each of the groups mentioned above which are responsible for serious diseases both in humans and animals.

### Pathogens that lyse and escape from the containing vacuole

#### Listeria monocytogenes

*L. monocytogenes* is a Gram-positive bacterium that causes a disease called listeriosis (Pamer, 2004; Lecuit, 2007) which involves severe gastroenteritis, infections, and central nervous system infections. This bacterium has the ability to lyse the vacuole after its entry into epithelial cells and macrophages. Subsequently, it replicates in the cytoplasm and spreads to other cells owing to an actin-based motility and formation of a secondary two-membrane vacuole in the neighboring cell, which is subsequently lysed.

*L. monocytogenes* secretes a pore-forming cytolysin called listeriolysin O (LLO) which plays an essential role in the escape step from the phagosome (Goldfine and Wadsworth, 2002; Kayal and Charbit, 2006). Secreted LLO inserts into phagosomal membranes and forms transmembrane pores that lead to phagosome disruption. This listeriolysin is encoded by the *hly* gene which is part of a virulence gene cluster (Dussurget et al., 2004; Scortti et al., 2007). The acidic environment of phagosomes (pH 5.5) is optimal for the action of LLO to pierce the phagosomal membrane.

Phospholipases also contribute to the escape of *Listeria* from the phagosome and play a key role in the disruption of the vacuolar membrane. *Listeria* secretes two C-type phospholipases, phosphatidylinositol-specific phospholipase C (PI-PLC) and phosphatidylcholine-specific phospholipase C (PC-PLC). These enzymes act specifically in the initial degradation of the inner membrane. It is believed that PI-PLC participates in the efficient lysis of primary vacuoles (Goldfine and Wadsworth, 2002) after direct invasion of host cells into a single membrane compartment. Subsequently, it is required the action of LLO which inserts into the vacuole membrane allowing access of PLC to the membrane leaflets. On the other hand PC-PLC participates in the rupture of the double membrane of secondary vacuoles where *Listeria* resides after infecting a neighboring cell. PC-PLC is activated through proteolytic cleavage by the bacterial acid-dependent metalloprotease Mpl, which also cleaves the actin nucleator ActA that is necessary for actin-mediated protrusion. After internalization by cell-to-cell spread, PC-PLC and PI-PLC participates in the dissolution of the inner membrane as they have preference for phosphatidylethanolamine, phosphatidylserine, and phosphatidylinositol lipid constituents of the inner leaflet. Acidification permits LLO secretion and perforation of the outer membrane. Then, PC-PLC completes the membrane dissolution as it also has preference for phosphatidylcholine lipids at the outer leaflet. Once in the cytoplasm, *Listeria* generates actin comets to move around and to infect neighboring cells by protruding at the plasma membrane.

#### Shigella flexneri

*S. flexneri* is a Gram-negative bacterial pathogen that causes dysentery, a disease called shigellosis that is manifested by severe diarrhea. During the early stages of infection, the bacteria are phagocytosed by macrophages and dendritic cells that are present in the follicle dome. Its intracellular fate includes escape from the phagocytic vacuole and induction of macrophage apoptosis.

*S. flexneri* utilizes a Type III (T3SS) secretion system to release protein effectors that manipulate the host cell. During the entry it secretes IpaB and IpaC that assemble into a complex (Blocker et al., 1999) within the host cell membrane to trigger bacterial uptake allowing, in addition, the translocation of other effectors (e.g., IpaA and IpgD) into the cytoplasm (Blocker et al., 2003). This complex also inserts into the vacuole membrane, thereby causing phagosomal lysis and escape into the cytoplasm. The *S. flexneri*-secreted protein IpaD facilitates the insertion of IpaB—IpaC pores into the membrane (Sansonetti et al., 1986; Blocker et al., 1999). The lysis of the vacuole is accompanied by the recruitment of cytosolic galectins to the remnant membranes after phagosome rupture (Paz et al., 2010). Once in the cytoplasm, another effector protein VirG (also called IcsA), an outer membrane protein, is gradually accumulated at one pole of the bacterium (Ogawa and Sasakawa, 2006). Vir G activates the nucleation of actin filaments during multiplication and is capable of interacting with N-WASP (a member of the Wiskott-Aldrich syndrome protein family), which is required for actin polymerization mediated by the Arp2/3 complex. VirA, another effector, was also recently identified to degrade tubulin. Thus, via the formation of actin tails *S. flexneri* propels through the cytoplasm moving towards the plasma membrane with VirA as a pivotal component creating a “tunnel.” *S. flexnerii* eventually moves through the host cell cytoplasm at the tight junctions and is phagocytosed by the neighboring cell, leading to intercellular spreading.

#### Mycobacterium marinum

*M. marinum* is a close relative of *M. tuberculosis* that causes a tuberculosis-like disease in fish. Both *M. tuberculosis* and *M. marinum* have a specialized type VII secretion system ESX-1 encoded by genes of RD1 (region of difference 1) and it is involved in the export of virulence proteins like ESAT-6, CFP-10, EspA, and Mh3881c. It has been shown that ESX-1plays an essential role in the escape of *M. marinum* from the *Mycobacterium*-containing vacuole (MCV) and also for lysis of the host cell plasma membrane (Abdallah et al., 2007; Xu et al., 2007). Secreted ESAT-6 may cause membrane pore formation in the MCV, facilitating *M. marinum* escape from the phagosome toward the cytoplasm where induces actin polymerization leading to bacterial motility and cell to cell spread. Actin polymerization requires the activation of host Arp2/3 by a member of the WASP family (Stamm et al., 2003; Smith et al., 2008). Also it has been shown that *M. marinum* and *M. tuberculosis* contain multiple copies of phospholipase C that could enhance membrane pore formation but the role of these enzymes requires further investigation. Interestingly, recent studies report that a fraction of *M. tuberculosis* and *M. leprae* escape from their phagosomal compartment and invade the cytosol in myeloid cells (van der wel et al., 2007).

*M. marinum* also modifies the composition of the phagosome. The association with the vacuolar H+-ATPase is undetectable at 6 h postinfection and during the proliferation phase the vacuole avoids the delivery of cathepsin D, a lysosomal protease, to the phagosomal lumen (Lerena and Colombo, 2011).

#### Group A streptococcus (GAS)

*Streptococcus pyogenes*, also known as Group A Streptococcus (GAS) is a common pathogen that causes a variety of acute infections including pharyngitis, skin infections, acute rheumatic fever, and life-threatening necrotizing fasciitis.

GAS enters non-phagocytic human cells via endocytosis and their phagosomes are labeled by EEA1, an early endosomal marker. GAS produces a variety of pathogenic factors such as streptolysin O (SLO), superantigens, and DNase. Bacterial cytolysin SLO is known to trigger multiple cellular responses, such as the induction of inflammatory cytokines and apoptosis but its principal function is to act as a cholesterol dependent pore forming cytolysin. The toxin inserts into the endosomal membrane thus, allowing GAS escape from the endosome-lysosomal pathway (Nakagawa et al., 2004). It is also known that SLO and LLO share 60% amino acid identity, and that their three-dimensional structures and characteristic domains are highly conserved.

Following bacterial escape into the cytoplasm, a population of GAS is captured and degraded by autophagy since the sequestering vacuoles acquire lysosomal enzymes leading to GAS degradation (Nakagawa et al., 2004). It is believed that the same SLO that promotes the bacterial escape from phagosomes also induces autophagy in host cells (please, see below).

### Pathogens that modify the phagosomal compartment

#### Legionella pneumophila

*L. pneumophila* is a facultative intracellular Gram-negative bacterial pathogen that in humans causes Legionnaires' disease that leads to a potentially lethal pneumonia following inhalation (Krech et al., 1980; Marra and Shuman, 1992; Xu et al., 2007). *L. pneumophila* enters to the cell via phagocytosis, and generates a phagosome that avoids the interaction with the endo/lysosomal pathway demonstrated by the lack of plasma membrane markers and endocytic markers on the *Legionella*-containing compartment (LCC) as early as 15 min after uptake (Clemens and Horwitz, 1992; Marra and Shuman, 1992). Instead, LCC is stained positive for ER-derived proteins like BIP and calnexin, the recombinant ER marker KDEL-YFP as well as mitochondria markers (Horwitz, 1983). Furthermore, the vacuoles are surrounded by double membranes studded with ribosomes and fuse with vesicles derived from the secretory pathway (Swanson and Isberg, 1995; Kagan and Roy, 2002) since it has been shown that at 1 h post-infection the LCC are decorated by the protein Rab1 and with the v-SNARE Sec22b (Derre and Isberg, 2004). *L. pneumophila* has numerous effector proteins which translocate into the host cell by the Dot/Icm system and modulate host GTPases that regulate membrane transport.

Interestingly, after several hours of infection, the LCC becomes acidic and is labeled by lysosomal markers, suggesting that at later infection stages it fuses with lysosomes (Sturgill-Koszycki and Swanson, 2000) where finally replicates and spreads cell to cell. It has been shown a bacterial flagellin induces pyroptosis in macrophages which leads to a host-derived pore forming activity, dependent on the Nlrc4 inflammasome with the consequence of host cell lysis and release of the bacteria.

#### Salmonella typhimurium

*S. typhimurium* is a Gram-negative bacterium that infects humans among other hosts and is a causative agent of gastroenteritis (Finlay and Brumell, 2000; Haraga et al., 2008). *S. typhimurium*, similar to S. *flexneri*, has a T3SS used to inject virulence factors into the host cells cytosol (Brumell et al., 1999).Some of these effectors promote the engulfment of the bacterium into a membrane bound compartment called the *Salmonella*-containing vacuole (SCV) that undergoes a maturation process (Brumell and Grinstein, 2004). The membrane of the SCV forms *Salmonella*-induced filaments (Sifs), which are long tubular structures necessary for bacteria replication (Garcia-del et al., 1993; Brumell et al., 2002; Birmingham et al., 2005). One effector protein SifA is indispensable for the formation of these structures and to manipulate the trafficking of the SCV (Beuzon et al., 2000; Brumell et al., 2001) inside the host cell. Elimination of the SifA function in manipulating the host trafficking machinery leads to vacuole disruption. A *Salmonella sifA* mutant rapidly becomes cytosolic, and its virulence is strongly attenuated in mice.

Early after internalization, the SCV transiently acquires the early endosome antigen-1 (EEA1) and the small GTPaseRab5. The *S. enterica* effector protein SopB is a phosphoinositide phosphatase that maintains Rab5 recruitment to arrest vacuole maturation. The vacuole then acquires late endosomal markers, such as the GTPaseRab7 and lysosomal-associated membrane protein 1(LAMP-1) (Brumell and Grinstein, 2004). However, it does not acquire mannose-6-phosphate receptor (M6PR) and cathepsin D suggesting that the SCV is not able to fuse with lysosomal compartments. These features allow the bacteria to rapidly replicate within the late SCV preventing their degradation in the lysosomal environment (Knodler and Steele-Mortimer, 2003; Brumell and Grinstein, 2004).

#### Mycobacterium tuberculosis

*M. tuberculosis* is an intracellular parasite that causes an infectious disease called tuberculosis (Lee et al., 1996). This bacterium survives in infected macrophages by blocking the maturation and biogenesis of the phagolysosome thus, the mycobacterial phagosomes do not acquire late endosomal and lysosomal characteristic (Armstrong and Hart, 1971; Russell et al., 2002).

*M. tuberculosis* produces several lipids, including species of phosphatidylinositol glycosilated. Many of these are factors involved in modulating phagosome maturation like LAM (lipoarabinomannan) that is believed to interfere with phagosomal acquisition of late endosomal markers (Fratti et al., 2001, 2003). On the other hand, PIM (phosphatidylinositol mannoside), which intercalates into host cell endomembranes (Beatty et al., 2000), stimulates homotypic early endosomal fusion and phagosome/early endosome fusion. *M. tuberculosis* also manipulates the recruitment and function of several Rab proteins, key molecules involved in vesicular transport.(Kyei et al., 2006; Roberts et al., 2006; Sun et al., 2007; Cardoso et al., 2010; Seto et al., 2011; Kasmapour et al., 2012). Thus, *M. tuberculosis* is a bacterium considered to belong to the group of pathogens that modify the phagosomal compartment. However, as mentioned above, it has been found that a fraction of *M. tuberculosis* and *M. leprae* can escape their compartment and invade the cytoplasm of the host (van der wel et al., 2007) and that a functional secretion system is required for a proper vacuolar escape. Indeed, it has been shown that the ESX-1 secretion system is responsible for the phagosomal membrane permeabilization. This allows the recognition of extracelular bacterial DNA and the access of the cytosolic components of the ubiquitin-mediated autophagy pathway. Recently, to investigate the phagosomal escape of *M. tuberculosis* to the cytosol a fluorescence resonance energy transfer (FRET) based method was used showing that *M. tuberculosis* ΔRD1 or BCG, both lacking the ESX-1 secreted protein ESAT-6 were unable to escape toward the cytoplasm (Simeone et al., 2012). This result points to a critical role of ESAT-6 in the escape mechanism.

Therefore, it is likely that *M. tuberculosis* uses more than one strategy to survive intracellularly, modifying the phagosome and also escaping toward the cytoplasm by lysing the containing-compartment.

### Pathogens that survive in acidic/degradative compartments

#### Coxiella burnetii

*C. burnetii* is an obligate intracellular bacterium that is the causative agent of Q-fever. It directs the biogenesis of a membrane-bound compartment called “parasitophorous vacuole” (PV) where the bacterium benefits of the acidic pH for its metabolic activation and resists the degradative function of the compartment.

*C. burnetii* PV transits the endolysosomal pathway to form a large phagolysosome (Voth and Heinzen, 2007). During the first minutes after uptake PV interacts with the early endosomal pathway as revealed by the presence of the markers Rab5 and EEA1. Later, the PV interacts with late endosomes as demonstrated by the recruitment of Rab7 (Romano et al., 2007) and with lysosomal compartments as it has been shown by the colocalization with other late endosome/lysosome markers such as LAMP-1, CD63, and M6PR.(Heinzen et al., 1996; Ghigo et al., 2002; Sauer et al., 2005; Shannon et al., 2005; Beare et al., 2009). In addition, the Coxiella PV contains active lysosomal hydrolases and cathepsin D showing that the maturing PV eventually fuses with lysosomal compartments, as evidenced also by acidification to pH 5.0 (Voth and Heinzen, 2007). This bacteria compartment also interacts with the autophagic pathway since it displays the autophagy protein LC3 on the limiting membrane even at very early times after infection (e.g., 5–10 min). Moreover, induction of autophagy favors Coxiella replication and development of the replicative vacuole (Gutierrez et al., 2005; Romano et al., 2007). Also it has been shown that the early secretory pathway contribute to the development of the CRV as demonstrated by the presence of Rab1 at its membrane. In addition, we have also demonstrated the participation of several SNAREs (Vamp3, Vamp7, and Vamp8) in homotypic and heterotypic fusion events in order to consolidate the replicative vacuole (Campoy et al., 2011, 2013).

*C. burnetii* actively manipulates PV biogenesis and other host cell processes for its successful replication. The PV requires bacterial protein synthesis for its maturation by the organism's Dot/Icm type IV secretion system (T4SS) since treatment with chloramphenicol, a bacterial protein synthesis inhibitor, impedes formation of the large and spacious PV (Howe et al., 2003). Inhibition of bacterial protein synthesis also hampers the recruitment of the protein LC3 and other critical factors required for the generation of the large replicative compartment (Romano et al., 2007).

## The autophagic pathway

### Molecular components

In eukaryotic cells three major types of autophagy have been described: macroautophagy, microautophagy, and chaperone-mediated autophagy (CMA). In microautophagy, portions of the cytoplasm are engulfed by invagination, protrusion and fission of the lysosomal membrane for a review see (Mijaljica and Devenish, 2011; Li et al., 2013). In contrast, in CMA unfolded and soluble proteins are translocated directly across the limiting membrane of the lysosome. On the other hand, macroautophagy is a conserved cellular degradation process in which portions of cytoplasm and organelles are sequestered into a double-membrane vesicle (i.e., autophagosome), and delivered by a vesicular transport event to a degradative organelle (i.e., the vacuole/lysosome), for breaking down and eventual recycling of the resulting macromolecules. In this review we will focus on macroautophagy, hereafter referred to as autophagy. The basic function of autophagy is in the turnover of long-lived proteins and the removal of protein aggregates or damaged organelles but it also has diverse roles in innate and adaptive immunity, such as resistance to pathogen invasion. Autophagy can be induced as a cellular response to various stress conditions, such as nutrient limitation and oxidative stress (Levine and Klionsky, 2004).

In mammalian cells, the first structural step in autophagy is the formation of the phagophores which are initiated as membrane curved prolongations called isolation membranes. These structures later become autophagosomes with a double membrane. The endoplasmic reticulum (ER) is believed to be involved in the origin of the phagophore although it has been demonstrated that other compartments also take part (Axe et al., 2008; Zoppino et al., 2010; Militello and Colombo, 2011). Subsequently, the autophagosomes undergo maturation into autolysosomes by fusion with endo/lysosomal organelles, the inner single membrane is released into the lumen and subsequently the captured cytoplasmic targets are degraded (Ohsumi, 2001; Levine and Klionsky, 2004; Klionsky, 2007; Mizushima et al., 2008).

There are a number of signaling complexes and pathways involved in the regulation of the initiation and maturation of autophagy. Also several autophagy-related (*ATG*) genes essential to drive this cellular process have been identified, both for selective and nonselective types of autophagy. Identification of the *ATG* genes in yeast, and the finding of orthologs in other organisms, has revealed the conservation of the autophagic machinery in all eukaryotes.

**Figure 1:**
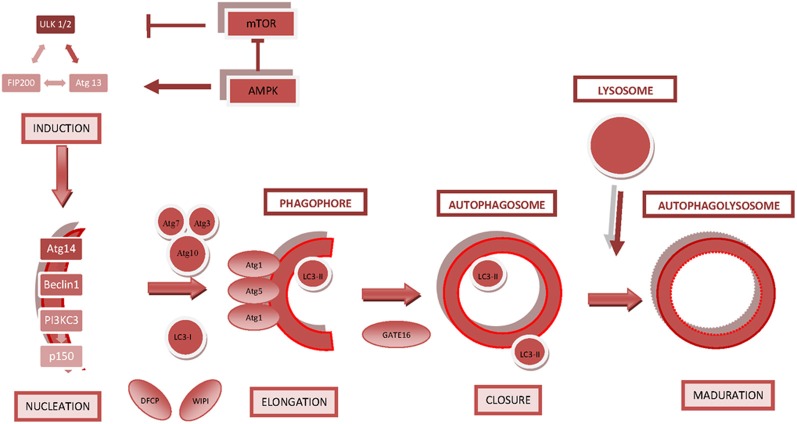
**Key regulatory molecules involved in autophagosome formation.** The diagram shows the steps that lead to the formation and closure of the autophagosome and subsequent maturation into an autophagolysosome/autolysosome. The main regulatory molecules and signaling complexes involved in autophagosome formation are depicted.

The core proteins and complexes that constitute the autophagic machinery and their main roles in autophagy are described below. (Figure 1).

#### mTOR

The protein mTOR (mammalian target of rapamycin) is a serine/threonine kinase highly conserved, involved in several regulatory pathways that acts as a sensor of nutrient or energy status and growth factors. mTOR regulates autophagy induction playing an inhibitory role in this process (Carrera, 2004). mTOR exists in two distinct complexes, mTORC1 and mTORC2 that are conserved from yeast to mammals (Loewith et al., 2002), mTORC1 is inhibited upon nutrient deprivation or by rapamycin allowing an increase in autophagic activity (Noda and Ohsumi, 1998). The mTOR kinase may inhibit autophagy through two general mechanisms: (i) by regulating Atg proteins, in a direct or indirect manner, thus, avoiding the formation of autophagosomes. (ii) by acting in a signal transduction cascade through various downstream effectors to control both translation and transcription.

***ULK complexes.*** In mammals, ULK1 and ULK2 (UNC- 51-like kinases) are the homologs of the yeast serine/threonine kinase Atg1 that plays a key role in the induction of autophagy, acting downstream of TORC1(Klionsky, 2005; Chan et al., 2007).These proteins, in a complex with Atg13 and Atg101 and focal adhesion kinase family interacting protein of 200 kDa (FIP200, a functional ortholog of Atg17), are essential for autophagosome formation in mammals (Hosokawa et al., 2009a,b; Mercer et al., 2009).ULK and FIP200 form a complex that is required during an early step in autophagosome formation. Atg13 directly interacts with ULK1, ULK2, and FIP200 independent of its phosphorylation state and FIP 200 binds to ULK1 and ULK2 independently of the nutrient status (Hara et al., 2008; Jung et al., 2009).The ULK1–Atg13–FIP200 complex contains mTORC1 and is constantly assembled and, upon autophagy induction is recruited to the phagophore. Under basal conditions, this complex interacts directly with mTOR, inhibiting ULK1/2 kinase activity. This latter regulates the formation of autophagosome and association of some Atgs with the phagophore. Under nutrient starvation, mTORC1 is dissociated from the ULK1 complex, with the resulting ULK1/2 dephosphorylation and consequent phosphorylation of FIP200, Atg13, and itself (Hosokawa et al., 2009a,b).

***Phosphatidylinositol 3-Kinase complexes.*** In mammalian cells, there are 3 classes of phosphatidylinositol 3-Kinases (PI3K): (i) Class I PI3K (protein kinase B, PKB, also known as Akt) is an inhibitor of autophagy. (ii) Class II PI3K activity is thought to have no relevance to autophagy control. (iii) Class III PI3K, a functional ortholog of yeast Vps34 (vacuolar protein sorting 34), is an activator of autophagy, presumably acting downstream of mTOR. In mammals, the formation of class III PtdIns3Kcomplex is conserved. Vps34 interacts with Vps15 (also called p150) and with Beclin1, the ortholog of Vps30/Atg6 (Panaretou et al., 1997; Liang et al., 2008). The Beclin 1/PI3K-III complex plays a crucial role at an early step of autophagosome formation in mammalian cells. Beclin1 localizes to the TGN (*trans*-Golgi network), the mitochondria, the perinuclear membrane and the ER and it exists in functionally distinct hVps34-containing protein complexes, including several modifier components such as Atg14L or Barkor (the ortholog of Atg14), which plays a role in initiation and UVRAG (ultraviolet irradiation resistance-associated gene) the ortholog of Vps38, which enhances the activity of Vps34 by stabilizing the association of Beclin1 and Vps34.

***Ubiquitin-Like Protein Conjugation systems.*** Autophagosome formation is believed to be driven by two protein-protein and protein-lipid conjugation systems that include the ubiquitin-like proteins Atg12 and Atg8. Both conjugation systems are evolutionarily conserved from yeast to humans The first system yields an Atg5-Atg12 covalent conjugate that associates non covalently with Atg16L1 (the mammalian equivalent of yeast Atg16) directing the site of the formation of the second protein-lipid conjugate. The second system yields LC3-II (Atg8-PE), which assists in autophagic membrane growth (Mizushima et al., 1998; Mizushima, 2002; Hanada et al., 2007).

– **Atg12-Atg5 Conjugation System:** Atg12 is covalently attached to Atg5 through an isopeptide bond between a C-terminal glycine of Atg12 and an internal lysine residue of Atg5. The conjugation reaction is catalyzed by two additional proteins, Atg7 and Atg10.Atg16, a coil-coil protein, binds preferentially to the Atg12-Atg5 conjugate. Atg16L links Atg12-Atg5 through self-oligomerization forming an Atg12-Atg5-Atg16L multimeric structure. This is necessary for the elongation of the phagophores and to specify the site of LC3 lipidation. This complex is not present in the mature autophagosome because it dissociates from the membrane after the formation of the autophagosome, thus, serving as a specific phagophore marker (Fujita et al., 2008a,b).
– **LC3 II (Atg8-PE):**LC3, the mammalian homolog of Atg8, is conjugated to a membrane lipid, phosphatidylethanolamine (PE) and it is present in the early isolation membranes and autophagosomes. Atg4, a cysteine protease, is responsible for processing pro-LC3 by cleaving a single Arg residue, consequently exposing Gly in its C-terminus. LC3 can be activated by Atg7 (E1) in an ATP-dependent manner and transferred to a conjugating E2 enzyme, Atg3. In a final step, LC3 is conjugated to PE through an amide bond between the C-terminal glycine and the amino group of PE. LC3-PE is tightly associated with membranes, being an integral membrane protein present in autophagosomes and it is utilized as a marker to monitor its formation as well as the activity of autophagy. Unlike the Atg12-Atg5 conjugate, LC3-PE conjugation is a reversible process in which Atg4 liberates LC3 from its target lipid. The released LC3 is recycled and used in another conjugation reaction to allow efficient progression of autophagy.
  There are multiple mammalian Atg8 like proteins, divided into two different subfamilies: LC3s and γ -aminobutyric acid receptor associated proteins (GABARAPs), all associated with autophagosomes. These subfamilies function at different steps of this process, LC3 mediates the elongations of the autophagic membrane and GABARAPs participates in the dissociation of the Atg12-Atg5-Atg16L complex. Among them, LC3 is most abundant in autophagosomal membranes and is well established as a marker to monitor the autophagosome and autophagic activity. The relative amount of membrane-bound LC3-II in general reflects the abundance of autophagosomes.

### Autophagy regulation

Autophagy is a highly regulated process that plays an important role in cellular homeostasis under basal levels through the elimination of damaged organelles as well as the turnover of long-lived proteins. On the other hand, under stress conditions, such as nutrient starvation, hypoxia, oxidative stress, pathogen infection, the level of autophagy elevates as a response, resulting in adaptation and survival; however, deregulated or excessive autophagy may lead to cell death (Yoshimori, 2004). Thus, defective autophagy has been implicated in the pathogenesis of diverse diseases, such as certain types of neuronal degeneration and cancer, and also in aging. More recently, in epithelial cells from cystic fibrosis (CF) patients, characterized by a dysfunctional CF trans-membrane conductance regulator (CFTR), a marked inhibition of autophagy has been observed (Luciani et al., 2010). Therefore, CF is considered a disorder with defective autophagy which is believed to contribute to the pathogenesis of the CF.

The regulation of the autophagic activity to prevent an unbalanced situation can be controlled by several complexes. As mentioned above, mTOR is a kinase that acts as a central sensor of growth factors, nutrient signals, and energy status and serves as a master regulator of autophagy. mTORC1 downregulates autophagy through the class I PI3K-protein kinase B (PKB, also known as Akt) pathway. Thus, the PI3K-I/PKB pathway is involved in the negative modulation of autophagy and it functions at the plasma membrane.Class I PtdIns3K is activated by activated receptor tyrosine kinases that autophosphorylate upon association with growth factors leading to an activation of PKB that phosphorylates a downstream protein complex, TSC2, activating mTORC1 (Vanhaesebroeck and Alessi, 2000; Brazil and Hemmings, 2001; Axe et al., 2008).

Apart from being a nutrient sensor, mTOR can also sense changes in the cellular energy levels via AMPK (AMP-activated protein kinase) (Noda and Ohsumi, 1998).AMPK is a sensor of hypoxia bioenergetics, and is activated by a decreased ATP/AMP ratio produced during nutrient and energy depletion. Active AMPK leads to phosphorylation and activation of TSC1/TSC2 thus, inhibiting mTORC1 activity (Hoyer-Hansen and Jaattela, 2007). On the other hand, AMPK also inactivates mTORC1 by direct phosphorylation of the mTOR binding partner raptor which is important for the inhibition of mTOR (Gwinn et al., 2008).In addition, AMPK directly phosphorylates and activates the complex ULK1/2, resulting in the activation of autophagy (Kim et al., 2011).

Beclin 1/hVps34 complex contributes to autophagosome formation by allowing other Atg proteins to relocate to the pre-autophagosomal structure. Beclin 1 acts as a platform, recruiting activators or repressors of Beclin 1/hVps34-dependent autophagy and the whole complex can be modulated by negative or positive regulators. Beclin1 was initially identified as a binding partner of the anti-apoptotic protein Bcl-2, an interaction that inhibits autophagy by preventing the association of Beclin1 with Vps34. Bcl-2 proteins do not directly compete with Vps34 for binding to Beclin 1, since they bind to the Bcl-2 binding domain of Beclin 1, whereas Vps34 is thought to bind to its EC domain. Hence, the binding of Bcl-2 to Beclin1 is tightly regulated and widely exploited by pathogens and by other regulatory processes. On the other hand, several proteins have been discovered to be active components of the pro-autophagic multimolecular complex: (i) UVRAG and Beclin 1 interact directly through their coiled-coil domain (Itakura et al., 2008; Liang et al., 2008) and is negatively regulated by Rubicon. (ii) AMBRA1 (the Activating Molecule in Beclin 1-Regulated Autophagy) stabilizes the association of Vps34 with Beclin 1(Fimia et al., 2007). (iii) The small GTPase Rab5, a regulator of early endocytosis, also interacts with and activates Vps34 in its complex with Beclin 1 and increases autophagosome formation. The PI3P generated by the Beclin 1/hVps34 complex allows the recruitment of Wipi (Atg18) via a PH domain and DFCP1 via a FYVE domain (Itakura and Mizushima, 2010; Matsunaga et al., 2010), which are important players in autophagosome formation.

It is important to take into account that a significant crosstalk between apoptosis and autophagy exits. As explained above the interaction between Bcl-2and Beclin1is key for the switch between autophagy and apoptosis. However, proteins that were originally thought to belong to the autophagic pathway have been found to modulate apoptosis either by inhibiting or inducing this type of cell death. For example ATG5, that plays an essential role in inducing autophagy, also functions as a pro-apoptotic protein when is cleaved by calpain (Yousefi et al., 2006).

As indicated above, autophagy is defective in epithelial cells from CF patients (Luciani et al., 2010). Interestingly, as a consequence of mutations on the CFTR the conformational defective protein leads to autophagy inhibition through the sequestration of Beclin 1 into aggresomes mediated by a ROS-transglutaminase complex [for a review see Villella et al. (2013)]. In addition, these autophagy deficient cells also accumulate the ubiquitin binding protein p62 (please see below), causing proteosome overload and aggresome formation contributing to hamper the clearance of the misfolded proteins. This situation generates a vicious loop with impeded autophagy and proteosomal degradation.

## Xenophagy: antibacterial autophagy

### Selective recognition of bacterial pathogens by autophagy: molecular machinery involved

Autophagic adaptors are proteins that participate in the recognition of pathogens by the autophagic machinery. These adaptors interact with both, components of the autophagic machinery and specific cargos targeted for autophagy degradation (Bjorkoy et al., 2005; Kirkin et al., 2009).The adaptor proteins binds both ubiquitin by means of an ubiquitin-associated (UBA) domain and ATG8/LC3 via an identified WXXL-like sequences known as AIM (Atg8-family interacting motif) and LIR (LC3 interacting domain region), respectively. LIRs consist of a beta-strand containing the WxxL motif that forms an intermolecular beta-sheet with Atg8/LC3 (Noda et al., 2010). These motifs are present in the protein p62 and neighbor of BRCA1 gene1 (NBR1) involved in autophagic degradation of protein aggregates, as well as in Atg32 and Nix required for mitophagy and in Atg19 for the cytoplasm-to-vacuole targeting pathway, linking the cargo to the autophagosome generating machineries [for a revision see Noda et al. (2010)].

As mentioned above, p62 (also known as SQSTM1) is an adaptor protein with multiple protein-protein interaction domains, including an UBA domain for binding to ubiquitinated cargo and a LIR domain for binding to LC3.The protein p62 was initially known as a molecular adaptor involved in the recognition of toxic aggregates to be deliver to the autophagic pathway (Bjorkoy et al., 2005; van der vaart et al., 2008; Komatsu and Ichimura, 2010). However, soon a wider role was uncovered demonstrating its participation in the detection of intracellular pathogens, as a molecular component responsible for the targeting of certain microorganism toward the autophagy pathway, revealing a novel function for p62 in innate immunity. Thus, the cell uses a conserved pathway for both the surveillance of misfolded proteins and intracellular bacteria.

*Salmonella enterica* serovar Typhimurium (*S. Typhimurium*) is known to manipulate the fate of its SCV by injecting effector proteins into the host cytoplasm via its Type III secretion system (Hueck, 1998; Knodler and Steele-Mortimer, 2003), and (Steele-Mortimer et al., 2002; Knodler et al., 2003). However, a small population of Salmonella that is unable to establish a stable SCV, is released into the cytoplasm after damaging its vacuolar niche. Upon entry into the mammalian cytosol the bacteria become decorated by a layer of polyubiquitinated proteins (Perrin et al., 2004; Birmingham and Brumell, 2006). It was shown that p62 is recruited to *S. typhimurium* and associates with ubiquitinated proteins localized to the bacteria (Zheng et al., 2009).Expression of p62 is required for efficient autophagy of the pathogen and for the control of bacteria intracellular replication. Interestingly, p62 is not the only adaptor protein to recognize intracellular Salmonella. The ubiquitin-coated cytosolic bacteria are also recognized by NDP52 (nuclear dot protein 52 kDa), an adaptor protein that binds both ubiquitin and LC3, which in turn, targets them for autophagy contributing to innate immunity (Thurston et al., 2009).NDP52 by binding the adaptor proteins Nap1 and Sintbad, recruits TBK1 (Tank-binding kinase 1)an IKK family kinase. Knockdown of NDP52 and TBK1 facilitates bacterial proliferation and impairs autophagy of Salmonella (Thurston et al., 2009). Interestingly, it has been shown that the two adaptor proteins, p62 and NDP52, are recruited at the same time (i.e. 60–90 min post infection) to bacteria-associated microdomains independently of each other and that both are required for antibacterial autophagy of *Salmonella enterica* (Cemma et al., 2011).Of note, double knockdown of both effectors do not have an additive effect on autophagy impairment indicating that they function in the same pathway and are not redundant (Cemma et al., 2011).Other pathogens that occasionally invade the cytoplasm such as *Streptococcus pyogenes* are also restricted by NDP52 and TBK1. Recently, the mechanism by which TBK1 restricts bacterial proliferation has been uncovered. Bacterial wall components such as lipopolysaccharide (LPS) activates TBK1 via the Toll-like receptor 4 (TLR4), a pattern recognition receptor (Wild et al., 2011).The activated TBK1 phosphorylates another autophagic receptor, optineurin (OPTN) which in turn binds to LC3. Indeed, TBK1-mediated phosphorylation of OPTN at Ser177 increases its affinity for LC3, targeting ubiquitin-coated cytosolic Salmonella to autophagosomes and favoring its elimination by autophagy. As expected, silencing of OPTN leads to Salmonella proliferation confirming the requirement for OPTN for bacterial restriction by autophagy (Wild et al., 2011). A role for TBK-1 in the autophagic elimination of *Mycobacterium tuberculosis* var. bovis BCG has been also recently reported (Pilli et al., 2012). It was shown that TBK-1 coordinated the assembly and function of the autophagic machinery and phosphorylated the autophagic adaptor p62.

**Figure 2:**
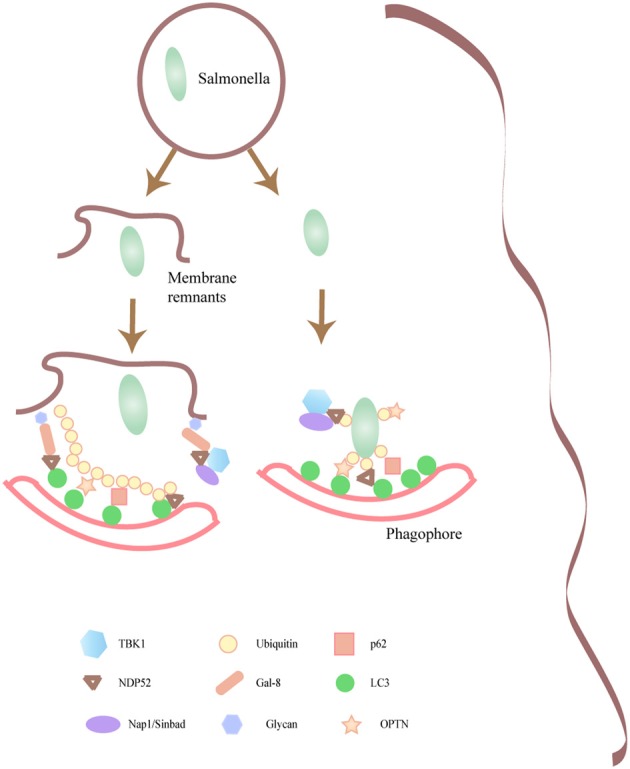
**Intracellular pathogens that damage the containing phagosomal membrane are targeted by autophagy.** Molecular components responsible for the targeting of certain microorganisms toward the autophagy pathway are depicted. Several of the autophagic adaptors such as p62, NDP52, that participate in the recognition of Salmonella by the autophagic machinery are shown.

Interestingly, OPTN colocalized with TBK1 and NDP52 but not with p62 on the surface of Salmonella that has escaped to the cytoplasm, supporting the idea that several molecular mechanisms participate in bacteria clearance (Weidberg and Elazar, 2011). In a recent publication it was shown that the cytosolic lectin galectin 8 detects damaged pathogen-containing vacuoles. Galectin 8 mediates the recruitment of NDP52 at early infection times followed by an ubiquitin-dependent NDP52 recruitment (Thurston et al., 2012).Thus, this study highlights the recruitment of NDP52 mediated by two different molecules in a sequential fashion to ensure the proper clearance of cytoplasmic Salmonella. (Figure 2).

It was expected that those intruders that are well-adapted to thrive in the cytoplasm such as *Shigella flexneri* were not restricted by NDP52. Indeed, in a previous publication *S. flexneri* was indicated not to colocalize with ubiquitin or NDP52 (Thurston et al., 2009) and consistently, NDP52 depletion did not affect *S. flexneri* proliferation. However, more recently Pascal Cossart and collaborators have shown that p62 and NDP52 target Shigella to an autophagy pathway dependent upon actin and septin, a newly characterized component of the cytoskeleton. Septin filaments form a cage in a myosin-dependent manner which traps cytosolic Shigella leading to bacteria restriction by autophagy (Mostowy et al., 2011). Indeed, the formation of septin cages wrapping bacteria that colonize the cytosol occurred concurrently with the acquisition of autophagy markers (Mostowy et al., 2010). In contrast, the Listeria ActA mutant is targeted to an autophagy pathway by p62 or NDP52 but independently of septin or actin (Mostowy et al., 2011). Thus, it seems that the proteins p62 and NDP52 drive intracytoplasmic Shigella and Listeria to different autophagic pathways.

In a study by Ogawa and collaborators (Ogawa et al., 2011), another autophagy cargo receptor has been identified but in this case the binding partner is not LC3 but upstream molecular components of the pathway. Tecpr1 (Tectonin domain-containing protein) binds Atg5 and WIPI-2, a PI(3)P-interacting protein involved in phagophore formation. Tecpr1, colocalized with Atg5 at Shigella-containing phagophores and its activity is necessary for efficient autophagic targeting of bacteria. Interestingly, Tecpr1 is also involved in autophagy clearance of protein aggregates and damaged mitochondria, but it is not required for canonical autophagy induced by starvation or rapamycin (Ogawa and Sasakawa, 2011).

In addition to ubiquitin, a role for diacylglycerol (DAG)-dependent signaling cascade in antibacterial autophagy has been described (Shahnazari et al., 2010). In this report the authors have shown that DAG production was necessary for efficient autophagy of Salmonella. DAG was localized to bacteria-containing phagosomes and preceded the recruitment of autophagic markers. Of note, the Salmonella-containing autophagosomes colocalized independently with either ubiquitin or DAG, indicating that the DAG-signaling pathway is an alternative mechanism to control autophagic clearance of bacteria.

Another signaling molecule that has an important role in both phagosome and autophagosome formation and maturation is ROS (Reactive oxygen species). Sources of ROS are, among others, mitochondria, ER, and peroxisomes. In phagocytic cells an important source of ROS is the NADPH oxidase associated with the phagosome which has a key function as a microbicidal molecule (Babior, 2000; Bylund et al., 2010; Kotsias et al., 2013) DAG is an upstream activator of the NADPH oxidase and ROS production is also required for LC3 recruitment to a self-tailored niche containing *L. monocytogenes* (Lam et al., 2013); please also see below next section. Interestingly, ROS actively regulates autophagy by targeting autophagy gene products such as the protein Atg4, the hypoxia inducible transcription facto Hif-1a and also the kinases mTOR and MAPKs (mitogen-activated protein kinases). In contrast, ROS production can be controlled via the removal of mitochondria by autophagy (i.e., mitophagy) (for a comprehensive review see Vernon and Tang, 2013).

One important question is how this autophagic process is initiated, since the xenophagic event commonly takes places in cells incubated in full nutrient condition. A critical role for amino acid and mTOR signaling modulation was recently uncovered. In a very recent study, it was demonstrated that infection with Shigella and Salmonella triggered an early state of intracellular amino acid starvation causing the dissociation of MTOR from endomembranes and the downregulation of its activity (Tattoli et al., 2012). Of note, this amino acid starvation signal was caused by host membrane damage, which was differentially elicited by Salmonella (a transient response) in contrast to the sustained starvation signal in Shigella-infected cells.

### Evasion from autophagy recognition

If autophagy is such a powerful defense mechanism as described above, how is it possible that certain pathogens have been able to adapt to survive and defend against this weapon machinery? What molecular mechanism are they using to be protected? In this review we will present same examples of how intracellular pathogens of either a vesicular or cytoplasmic lifestyle evade autophagy recognition.

A very interesting autophagy escape mechanism has been unveiled in the case of *S. flexneri*. Soon after internalization *Shigella* disrupt and break out from the containing membrane vacuole toward the cytoplasm where the bacteria active multiply and move infecting neighboring cells. This movement is carried out by directing local actin polymerization at one pole of the bacterium which is dependent on the exposure and accumulation of the bacterial protein VirG (Goldberg et al., 1993).VirG interacts with N-WASP which in turn via the Arp2/3 complex induces actin polymerization. A study revealed that VirG, via interaction with the autophagy protein Atg5, is a key molecule in the targeting of cytoplasmic *Shigella* by autophagy (Ogawa et al., 2005). IcsB an effector protein secreted by *Shigella* via the T3SS (type three secretion system) is capable of binding VirG inhibiting competitively the binding of Atg5 to VirG, preventing autophagy targeting of the bacterium. In contrast, in the *icsB* mutant, Atg5 binds to the VirG and cytoplasmic *Shigella* are recognized by autophagy being trapped in acidic LC3-positive compartments. As a consequence the *icsB* mutant presents a phenotype characterized by deficient ability to multiply intracellularly. Thus, the binding of IcsB to VirG which prevents the interaction with Atg5 at one pole of the bacterium would serve as a shield protecting *Shigella* against autophagic recognition. Interestingly, it has been recently shown that *Shigella* uses another device to escape from autophagy (Dong et al., 2012). The VirA effector exhibits potent RabGAP activity and specifically inactivates the Rab protein, Rab1 disrupting ER-to-Golgi trafficking. Rab1 has been shown to be required for autophagosome formation (Zoppino et al., 2010) thus, via VirA *Shigella* negatively modulates the autophagic pathway avoiding this pathogen clearance mechanism.

*Listeria monocytogenes* also uses an actin-based motility to escape from autophagy. ActA is the bacterial protein responsible for actin polymerization once the bacterium has reach the cytoplasm. It has been shown that a non-motile *actA* mutant of *L. monocytogenes* treated with the bacteriostatic antibiotic chloramphenicol is trapped by autophagy in the cytoplasm of infected macrophages (Rich et al., 2003). Although, ActA expression was sufficient to prevent bacteria targeting by autophagy in the cytoplasm of macrophages, ActA expression was not strictly necessary since an *actA* mutant was competent to evade autophagy in the absence of chloramphenicol treatment, indicating that other virulence factors were involved (Birmingham et al., 2007). It was found that the bacterial phospholipases, PI-PLC, and PC-PLC play an important role in autophagy evasion. More recently Yoshikawa and collaborators have demonstrated that recruitment to the bacterial surface of the Arp2/3 complex and Ena/VASP, mediated by ActA, camouflages the bacteria from autophagic recognition, regardless of the bacterial capacity for movement. Thus, an ActA mutant bacteria unable to recruit these actin nucleating proteins were ubiquitylated and subsequently, recruited p62 and LC3, undergoing autophagy (Yoshikawa et al., 2009a,b).

**Figure 3:**
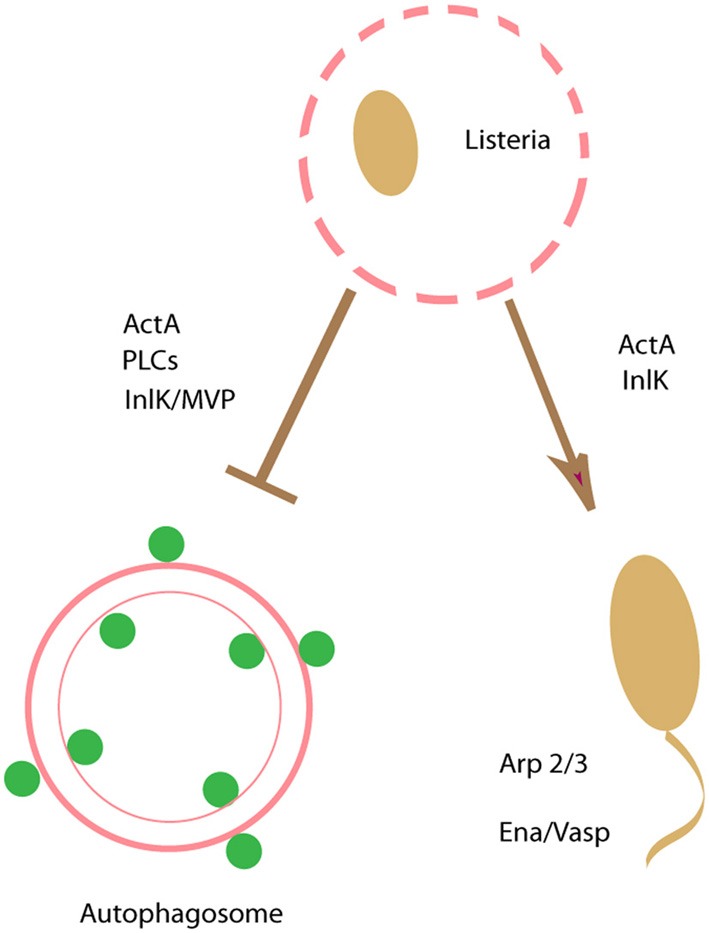
**Escaping from autophagy recognition.** *Listeria monocytogenes* uses an actin-based motility to escape from autophagy.ActAand PLCs are key bacterial factors to prevent bacteria targeting by autophagy. Recruitment to the bacterial surface of the actin nucleating proteins Arp2/3 and Ena/VASP, mediated by ActA, camouflages the bacteria from autophagic recognition. In addition, a member of the internalin family of proteins, InlK, a protein expressed at the surface of *Listeria*, and its host partner the Major Vault Protein (MVP) prevents *L. monocytogenes* from autophagic recognition.

Interestingly, *Listeria* employs another strategy to avoid autophagy. Internalins are a family of proteins mostly expressed at the surface of *Listeria* (Glaser et al., 2001). The role of one member of this family, InlK was recently demonstrated as a virulence factor important for bacteria infection *in vivo* (Dortet et al., 2011). The Major Vault Protein (MVP) was identified as a host partner of InlK. The interaction between InlK/MVP takes place at the bacterial surface in the cytoplasm of infected cells independently of actin polymerization. Of note, it was found that the recruitment of MVP prevents *L. monocytogenes* from autophagic recognition, leading to enhanced bacterial survival in infected cells. Thus, *L. monocytogenes* utilizes more than one mechanism to avoid targeting by the autophagic pathway during colonization of host cells (Figure 3). Of note, a population of *L. monocytogenes* that seems to remain in the phagosome forms Spacious *Listeria*-containing Phagosomes (SLAPs), which are large endosome- or autophagosome-like compartments containing multiple bacteria. These self-tailored compartments do not mature but require bacterial LLO and host autophagy to form. *L. monocytogenes* replicate in these phagosomes, though at a much slower rate compared to cytoplasmic bacteria and these compartments have been associated with persistent infection (Lam et al., 2013)

The Gram-negative tularemia-causing bacterium *Francisella tularensis* is a facultative intracellular pathogen capable of surviving and growing in several mammalian host cells. The bacteria are phagocytosed but escape the phagosome a few hours after infection (Clemens et al., 2004; Chong et al., 2008) actively multiplying in the cytoplasm although the pathogen seems to occupy different compartments during its intracellular cell cycle [for a revision see Chong and Celli (2010)]. Prior to escaping from the phagosome, *Francisella* initially resides within a vacuole that interacts with early and late endosomes but it does not recruit lysosomal markers (Clemens et al., 2004; Checroun et al., 2006; Chong et al., 2008; Santic et al., 2008; Wehrly et al., 2009). Regarding the relationship with the autophagic pathway apparently there is no evidence for an autophagic response, suggesting that the pathogen avoids its recognition by the autophagic machinery or it may inhibits the autophagic response. Indeed, it has been shown that several autophagy-related genes (ATG) are downregulated during infection of human monocytes suggesting that *Francisella* suppresses autophagy at the gene expression level (Butchar et al., 2008; Cremer et al., 2009). Interestingly, it has been shown in murine BMMs that a subpopulation of cytosolic *F. tularensis* is capable of reentering the endocytic pathway residing in a compartment with autophagosomal features by 24 h after infection (Checroun et al., 2006). In a recent publication it has been shown that a replication-deficient mutant is captured from the cytoplasm of murine and human macrophages into double-membrane vacuoles colocalizing with the late endosomal marker, LAMP1, and the autophagic protein LC3, limiting intracellular bacteria viability (Chong et al., 2008). These vacuoles were also labeled with ubiquitin and the autophagy adaptors p62 and NBR1. Thus, these findings suggest that Francisella avoids autophagic recognition to ensure bacterial growth in the host cells. Nevertheless, additional work is required to fully elucidate the complex interplay between Francisella and the autophagic pathway.

Similar to Francisella that suppresses autophagy by downregulating autophagy at the gene expression level, another bacterial pathogen *Burkholderia cenocepacia*, a pathogen that causes severe and persistent infections in CF patients, also downregulates the expression of critical autophagy genes such as Atg12, Atg5, and Atg8 (Abdulrahman et al., 2011). It is interesting to mention that autophagic dysfunction in infected cells is even more pronounced in CF macrophages compared to WT macrophages. This is consistent with the observation that epithelial cells from CF patients have a defective autophagic response even in non-infected cells as we have described above (Luciani et al., 2010). Thus, limited formation of autophagosomes in CF macrophages promotes *B. cenocepacia* survival. In contrast, autophagy stimulation by rapamycin treatment enhances the targeting of *B. cepacia* to the lysosomal compartment. (Abdulrahman et al., 2011; Li et al., 2013) In addition, it has been shown that *B. cepacia* inactivates the small GTPase Rab7 (Huynh et al., 2010), a key molecule required for the maturation and completion of the autophagic pathway (Jager et al., 2004; Gutierrez et al., 2005). Thus, inactivation of this Rab protein is part of the strategy used by *B. cepacia* to avoid autophagic clearance.

In a previous work we have shown that activation of autophagy by starvation or by other means (i.e., rapamycin-treatment) results in autophagic clearance of *Mycobacterium bovis* BCG and *M. tuberculosis* (Gutierrez et al., 2004). In addition, we have also shown that *M. marinum*, a fish pathogen that causes similar granulomas in the hands of infected humans to those caused by *M. tuberculosis* in the lungs, was able to induce the recruitment of LC3 to the *M. marinum*-containing phagosomes (Lerena and Colombo, 2011). However, these compartments were devoid of lysosomal enzymes indicating that fusion with lysosomes was prevented. Interestingly, this LC3 recruitment was dependent on a functional Esx-1 secretion system (Lerena and Colombo, 2011). Consistent with our results, in a recent publication Romagnoli and collaborators have found that the pathogenic *M. tuberculosis* strain Mtb H37Rv hampers the fusion of autophagosomes with lysosomes (Romagnoli et al., 2012). In contrast, the attenuated strains Mtb H37Ra or BCG, which are deficient in components of the ESX-1 secretion system (incompetent to secrete the protein ESAT-6) were unable to prevent autophagosome maturation. The ability to inhibit the autophagic flux was recovered in recombinant BCG and Mtb H37Ra strains in which the ESX-1 region was restored by genetic complementation. In another report it has been shown that ectopic expression of the ESAT-6/CFP-10 fusion in macrophages hampered autophagosome formation increasing *M. tuberculosis* viability. Interestingly, expression levels of ATG also diminished, suggesting that the fusion protein may modulate autophagy through the regulation of ATGs (Zhang et al., 2011). Of note, in a recent publication it was shown that phagosomal piercing mediated by the ESX-1 secretion system allows cytosolic components of the ubiquitin-mediated autophagy pathway access to phagosomal *M. tuberculosis* and also the recognition of extracellular bacterial DNA by the STING-dependent cytosolic pathway (Watson et al., 2012)

It has also been reported that the “enhanced intracellular survival” (eis) gene of *M. tuberculosis* plays essential roles in modulating autophagic and inflammatory responses in macrophages (Shin et al., 2010). Cells infected with an Mtb eis-deletion mutant H37Rv (Mtb- Δeis) presented a marked accumulation of autophagic vacuoles. In addition, the presence of this mutant bacterium in typical double-membrane autophagic structures was visualized by electron microscopy analysis. Thus, the protein eis seems to act as a negative regulator of autophagy contributing to avoid autophagic clearance of *M. tuberculosis*. In a recent publication Roy and collaborators have found that *Legionella* secretes via the Type IV secretion system an effector protein called RavZ (Choy et al., 2012).This factor disrupts the autophagy pathway by cleaving the C-terminal region of lipid-conjugated LC3/Atg8. Thus, *Legionella* inhibits autophagy in the host cell by direct manipulation of a key protein required for autophagosome formation.

## Subversion of autophagy taking advantage of the autophagic pathway

Several intracellular bacterial pathogens such as *Coxiella burnetii, Staphylococcus aureus, Legionella pneumophila*, *Anaplasma phagocytophilum, Brucella abortus*, have the ability to manipulate the autophagic pathway for their own benefit. These pathogens may stimulate their uptake into autophagosomes by the secretion of bacterial effectors (Amer and Swanson, 2005) In addition, these microorganisms appear to efficiently growth within autophagosome-like vacuoles. Indeed survival of some of these pathogens is reduced by autophagy inhibitors or in cells defective for essential autophagy genes such as Atg5 (Romano et al., 2007; Mestre and Colombo, 2012) Due to space limitations the mechanisms employed by these pathogens are not presented here, but these topics have been previously discussed in other reviews (Ogawa et al., 2005; Amer, 2013). In summary, in spite of acting as a critical component of the innate immune system to restrict some intracellular pathogens, autophagy can be modified or even exploited by certain microorganisms to favor pathogen survival and growth.

## Concluding remarks

As described in this review the autophagic pathway acts as a host cell effector mechanism to protect against pathogen invasion. However, many intracellular bacterial pathogens have developed highly advanced mechanisms not only to evade autophagic recognition but to manipulate the autophagic pathway for their own benefit likely by remodeling the autophagosomal compartment. While there is a general understanding on the overall survival strategies used for several of the microorganisms analyzed in this review, much uncertainty remains on specific aspects of how the autophagic response is triggered or how the pathogens escape and avoid autophagy clearance.

### Conflict of interest statement

The authors declare that the research was conducted in the absence of any commercial or financial relationships that could be construed as a potential conflict of interest.
